# Chromatic Sensor to Determine Oxygen Presence for Applications in Intelligent Packaging

**DOI:** 10.3390/s19214684

**Published:** 2019-10-28

**Authors:** Gracia López-Carballo, Virginia Muriel-Galet, Pilar Hernández-Muñoz, Rafael Gavara

**Affiliations:** Packaging Group, Instituto de Agroquímica y Tecnología de Alimentos, CSIC, Av. Agustin Escardino 7, 46980 Paterna, Spain; glopez@iata.csic.es (G.L.-C.); vmuriel@iata.csic.es (V.M.-G.); phernan@iata.csic.es (P.H.-M.)

**Keywords:** intelligent packaging, oxygen sensor, methylene Blue, light activation, retortable

## Abstract

A chromatic sensor has been designed for the detection of oxygen in package headspace. The sensor is based on the redox change of methylene blue (MB) to its leuco form. Its formulation includes the pigment, glycerol, as a sacrificial electron donor, TiO_2_, as a photocatalyst and ethylene-vinyl alcohol copolymer (EVOH), as a structural polymer matrix. The final sensor design that allows its manufacture by conventional printing and laminating technologies consists of the sensing polymer matrix (MB-EVOH) sandwiched in a suitable transparent multilayer structure. The outer layers protect the sensor from the external atmosphere and allow visualization of the colour. The inner layer is sufficiently opaque to facilitate sensor reading from the outside, is thick enough to avoid direct contact with food (functional barrier), and is oxygen-permeable to expose the sensing material to the internal package atmosphere. In the absence of oxygen, the sensor becomes white by irradiation with halogen lamps in less than 60 s. All components are substances permitted for food contact except the pigment, but specific migration analysis showed no trace of migration thanks to the functional barrier included in the design.

## 1. Introduction

There is increasing demand for food products with minimal processing, absence of additives, excellent sensory properties and long shelf-life. In response to this demand, novel processing and packaging technologies have been developed, and they are often combined to make possible the commercialization of pleasant, healthy, additive-free food. Many ready-to-eat foodstuffs are among these products, and they are put on the shelves with long shelf-life periods thanks to the maintenance of an oxygen-free atmosphere. Modified atmosphere packaging is an established packaging technology based on the positive effect of a specific gas mixture on food quality and safety stability. Among these gas mixtures, those without oxygen are often found in ready-to-eat products since oxygen is involved in microbial growth and oxidative processes, the two most common food deterioration processes. This is the case of baby foods, products without preservatives, reduced in salt and acid contents, and therefore, easily spoiled by growth of aerobic microorganisms or oxidative process in the presence of oxygen. It has been reported that aerobic bacteria and fungi grow at oxygen concentrations as low as 2% after a lag time of few days [1], but they are fully inhibited at 0.5% [2]. Recently, intelligent packaging systems, defined as those that monitor the condition of packaged food or the environment surrounding the food [3], have been developed to provide consumers with relevant information to help them to make their consumption decision. The intelligent packaging systems that are being studied are mainly based on temperature control, microbiological control, development of off-flavours or gas control.

Several studies have focused on the development of oxygen sensors, chemical systems that undergo a colour change when an environmental condition takes place. According to Mills [4], an oxygen sensor must be cheap, easy to interpret, non-toxic, sensitive to oxygen concentrations below 2% and irreversible, very stable and easy to include in the packaging system. With these requirements in mind, several pigments have been utilized, including natural pigments such as myoglobin [5,6], and synthetic dyes such as indigo or methylene blue [7]. This latter work reports on the preparation of an oxygen sensor based on a redox dye, methylene blue, a sacrificial electron donor, DL-threitol, and a semiconducting photocatalyst, TiO_2_ nanoparticles, all these components being supported on a polyethylene matrix. When the sensor was exposed to UV light, electrons from the photocatalyst were excited and oxidized the sacrificial electron donor. Then the methylene blue molecules took the free electrons and were reduced to a colourless form. The sensor, in the form of 70 µm thick film, was produced by extrusion and changed from dark blue to grey when exposed to UV light. Similar sensors have been reported with variations of sensor components: sacrificial donors (glycerol, DL-threitol, tartaric acid, triethanolamine), photocatalyst (micro- or nanoparticulated TiO_2_), supporting polymer (zein, cellulose, LDPE), dye (methylene blue, thionine) [8,9,10,11,12,13,14,15,16]. A scheme of the functioning process of the sensor is described in Section 3.2.

In this work, an oxygen sensor based on methylene blue was developed, characterized and tested for application in food packaging. In the design, the sensor is considered to accomplish the technical and legal requirements for this kind of intelligent packaging in terms of sensitivity (to the presence of low oxygen concentrations), irreversibility (in the presence of oxygen), stability (no colour change in anaerobic atmosphere), machinability, economics, non-toxicity and easy consumer interpretation (noticeable to the naked eye).

## 2. Materials and Methods

### 2.1. Materials

One-propanol was supplied by Panreac Química S.L.U. (Barcelona, Spain). Acetic acid, ethanol, methanol (HPLC) by Scharlau (Barcelona, Spain), methylene blue (MB) by Sigma (Madrid, Spain), glycerol by VWR International (Barcelona, Spain), micronized TiO_2_ by Guinama (Valencia, Spain). Thirty µm white polyethylene film was kindly provided by Huecograbados Fina (Valencia, Spain), 30 µm PET/SiOx/PE film by Tetra Laval Food (Bakel, Netherlands), 30 µm bioriented polypropylene BOPP by Envaflex (Zaragoza, Spain), and pellets of ethylene-vinyl alcohol copolymer EVOH (Soarnol 29 molar percentage of ethylene) by The Nippon Synthetic Chemical Company (Osaka, Japan). Distilled water was obtained by a Milli-Q Plus Millipore purification system (Molsheim, France).

### 2.2. Methods

#### 2.2.1. Sensor Preparation

EVOH is an extrudable thermoplastic commonly used in food packaging for its capacity to provide an oxygen barrier for flexible films and rigid containers. However, this copolymer was selected as the supporting polymer for its ability to be dissolved in hydro-alcoholic mixtures and applied by coating or printing procedures on flexible substrates. Examples of these applications in active packaging can be found elsewhere [17,18]. EVOH pellets were dissolved in a 1:1 (v/v) mixture of 1-propanol:water (PrOH/H_2_O) at concentrations ranging between 7 and 13% (w/v) under reflux at 75 °C and vigorous stirring. In parallel, TiO_2_ and glycerol were mixed in 1-propanol at room temperature with vigorous stirring for 15 min and a 30-min treatment in an Ultrasons ultrasonic bath (J.P. Selecta S.A., Barcelona, Spain). Methylene blue was also dissolved in water. Methylene blue and TiO_2_ solutions were mixed and added to the EVOH solution, and the final mixture was stirred for 15 min and ultrasonicated for an additional 30 min. The concentrations of the various components in relation to that of the dry EVOH, expressed in w/w percentages, were: from 7 to 100% of glycerol, from 7 to 100% of TiO_2_, from 0.2 to 2.5% of MB.

The final solution was used to prepare self-standing films and coatings. Self-standing films were obtained by spreading the solution on white PE film using a 100 µm coater bar (Lin Lab Rioja, Logroño, Spain). To improve wettability of the film surface, a previous treatment was made with a BD-20AC corona treater (Electro-Technic Products, Inc., Chicago, IL, USA). The films were dried by being placed in a home-made hot air tunnel consisting of 5 (500 W) Osram 64,700 halogen lamps and a fan (20 cm, 32 W, 1500 rpm) for 1 min. Coatings were prepared by spreading the film-forming solution onto corona-treated white (TiO_2_) polypropylene film. The final sensor film was obtained by coating 50 µm white PP film by flexography, followed by lamination of the resulting film to a PET-AlOx/PA structure (12/20 µm) by using a two-component polyurethane adhesive, at the Ducplast factory (Pobla del Duc, Spain). The final layer structure is schematically presented in Figure 2B.

#### 2.2.2. Rheology

Given that the manufacturing procedure should be easy to implement in common industrial plastic converting equipment, the viscosity of the final solution should be optimized to be applied like inks in gravure or flexography printing machines. Accordingly, the sensor-forming solution should have a viscosity of about 25 s measured with a no. 4 Ford cup (time required for 100 mL of solution to pass through an opening of 4 mm). To complete the rheological characterization of the solution, a Thermo Haake RheoStress 1 rotational viscometer (Thermo Fisher Scientific, Waltham, MA, USA) with a cone/plate system was used. The shear stress vs. shear rate data were collected and analysed with the equipment software.

#### 2.2.3. Morphology of the Sensor

The microstructural morphology of the sensor film was analysed by field emission scanning electron microscopy on the surface of a cryogenic fracture, following the procedure reported by Cerisuelo, et al. [19]. A digital image of the final sensor structure obtained industrially was acquired with an Eclipse 90i Nikon microscope (Nikon Corporation, Tokyo, Japan) with a 16× objective equipped with a digital camera (Nikon DS-5Mc, Nikon Corporation, Tokyo, Japan). Film was immersed in liquid N_2_ and cut. Image of the cut surface were captured, processed and analysed by the Nis Elements BR3.2 software (Nikon Corporation, Tokyo, Japan) software. The image was acquired using 40 x gain.

#### 2.2.4. Characterization of the Sensing Capacity

After the industrial production of the intelligent film, the activation of the sensor was tested with various lamps and exposure times to optimize the activation procedure. Film samples were inserted in 10 × 10 cm high-barrier PET-SiOx/PE bags under vacuum and exposed for increasing times to: a 6 W UV Xe excimer lamp at 172 nm (UV-Consulting Peschl España S.L., Valencia, Spain), a 75 W NIQ 80/36 U lamp (Heraeus, Boadilla del Monte, Spain) 253.7 nm or 500 W Osram 64,700 Tungsten halogen lamps (white light with highest intensity in the red spectral range). There were 4 Standard incandescent mercury lamps (blue coloured 100 W E27 230V A55, Philips, RS-Amidata, Pozuelo de Alarcón, Spain) also tested and discarded (no effect on sensor after 2 h). The activation time of the sensor is the minimum exposure that produces the colour change from blue to white.

Once the activation procedure had been selected, the sensing capacity was analysed. First, circular (10 mm in diameter) samples were inserted in 10 × 10 cm pouches of PET-SiOx/PE together with an OpTech^®^-O2 Platinum oxygen sensor and the pouches were vacuum sealed. The pouches were exposed to radiation to activate the sensors, and sensor colour and oxygen concentration were monitored with a Konica Minolta CM 3500d colourimeter (Aquateknica S.A., Valencia, Spain; following the methodology described elsewhere; [20] and an OpTech^®^-O2 Platinum Oxygen Analyzer (MOCON Inc., Minneapolis, MN, USA; [21], respectively. Colour data were recorded in CIELab* coordinates, L* (lightness value, 0 darkest black, 100 brightness white), a* (a* < 0 green, a* > 0 red) and b* (a* < 0 blue, a* > 0 yellow).

Sensors were also tested in bags containing an inert atmosphere. Four sensor samples and one OpTech sensor were vacuum packaged in BOPP pouches and immediately placed in 20 cm × 20 cm PET/SiOx/PE pouches which were sealed with a dry nitrogen atmosphere in a Multivac C350 (Multivac Packaging Systems, Sant Cebrià de Vallalta, Spain). The sensors were activated and sensor colour and oxygen concentration in the headspace were monitored.

#### 2.2.5. Test with a Real Food Product

Twenty g of infant milk powder was packed in 200 cm^2^ PET/SiOx/PET bags with N_2_ as an inert gas. Two sensors and an OpTech oxygen meter were adhered to the inside surface of each bag. The sensors were activated after packaging, and colour and oxygen were monitored using the same conditions as above. Various amounts of oxygen were injected through a septum. Both parameters were monitored during the first six hours following activation, followed by a daily measurement for up to one month. The experiment was carried out in duplicate, for 10 samples.

To check the evolution of the milk in these packages, an analysis of the hexanal concentration was carried out, as a parameter indicating the oxidation of the product [22]. For its quantification, a GC-MS/SPME technique was used. Specifically, hexanal extraction was carried out by inserting a CAR/PDMS (Supelco, Barcelona) solid phase microextraction fibre (SPME) into the bag through a self-adhesive septum. The extraction fibre was exposed for 60 min, and immediately inserted into the GC injector and desorbed for 5 min. The conditions of the Agilent Technologies (Barcelona, Spain) CG-MS model 5972 were: injector temperature 210 °C, splitless injection, capillary column of 30 m, 0.32 mm, 0.25 μm TRB-5MS (Teknokroma, Barcelona, Spain). Helium was the carrier gas, and the oven programme was 5 min at 40 °C, followed by a ramp up to 60 °C at 3 °C/min, up to 200 °C at 10 °C/min and maintained for 5 min at 200 °C. The hexanal was identified by comparison with the mass spectrum of the reference database (NIST 98), and by comparison with injection of the pure compound. Calibration was not performed, so integration areas of the corresponding peak were compared since they are proportional to the actual hexanal concentration.

#### 2.2.6. Methylene Blue Migration

Although methylene blue has several applications in medicine [23], this dye is not considered a food colourant or food additive, and therefore the specific migration of this substance is an important datum for its application in intelligent food packaging. Accordingly, several migration analyses were planned in this study. Bags measuring 10 × 10 cm were prepared with the intelligent film filled with 100 mL of various food simulants (3% acetic acid solution (v/v) as acid food simulant, and 10% ethanol solution (v/v) as aqueous non-acidic food simulant), heat-sealed, and stored for 10 days at 40 °C, following European regulations [24]. Also, bags filled with 100 mL of water were heated in boiling water for 15 min. Tests were carried out in triplicate. After exposure, the presence of MB in the simulants was analysed by measuring the absorbance at 660 nm in an Agilent 8453 UV-vis spectrometer (Agilent Technologies, Barcelona, Spain).

## 3. Results and Discussion

### 3.1. Rheological Analysis

As mentioned earlier, this work is focused on the preparation of an oxygen sensor that can be manufactured in industrial lines by conventional converting technologies. Since flexography is the most common printing process, one of the requirements of this development was to obtain an ink dispersion with appropriate viscosity, equivalent to 25 s in a no. 4 Ford cup. Since the ink component most affecting the viscosity is the polymer, followed by the addition of the TiO_2_ nanoparticles, several solutions were prepared with polymer concentrations ranging from 7 to 13% (w/v of solvent), and TiO_2_ ranging from 25 to 100% (w/w of dry polymer). Table 1 summarizes the results obtained with the Ford cup, which indicate that the appropriate polymer concentration to obtain a varnish adequate for flexography would be 7%, the effect of the addition of the inorganic load being less relevant in terms of viscosity.

In order to better understand the rheological behaviour of the product, the shear stress (σ in Pa) of the above solutions and dispersions was analysed as a function of the shear rate ($\dot{y}$ in 1/s). According to the Ostwald–de Waele law:

(1) $$\sigma =K\cdot {\dot{y}}^{n}$$

where ***K*** (in Pa·s^n^) is the consistency index and ***n***, the flow behaviour index, expresses the type of fluid analysed: ***n*** = 1 indicates Newtonian fluid, ***n*** < 1 pseudoplastic fluid, and ***n*** > 1 dilatant fluid.

The raw data are represented in Figures S1 and S2 in the Supplementary Materials. From the fitting of experimental data to the Ostwald–de Waele equation, data of ***K*** and ***n*** were obtained and are included in Table 2. As can be seen, the EVOH solutions behaved like Newtonian fluids, with ***n*** values approaching 1 and without significant differences within the concentration range tested. Obviously, the viscosity increases with the polymer concentration, which is reflected in the increase in the consistency index.

The addition of TiO_2_ modified the viscosity of the EVOH solutions slightly. As Table 2 (and Figure S2) shows, the viscosity increases as a consequence of an increase in the consistency index. However, the ***K*** values did not present significant differences with the concentration of the oxide. With respect to the ***n*** value, dispersions containing TiO_2_ presented values close to 1, but with a significant decrease compared to the ***n*** values of neat EVOH solutions. These values indicate that the dispersions might present a slightly pseudoplastic behaviour, typical of complex polymer solutions and dispersions.

### 3.2. Film Preparation

**Figure 1:**
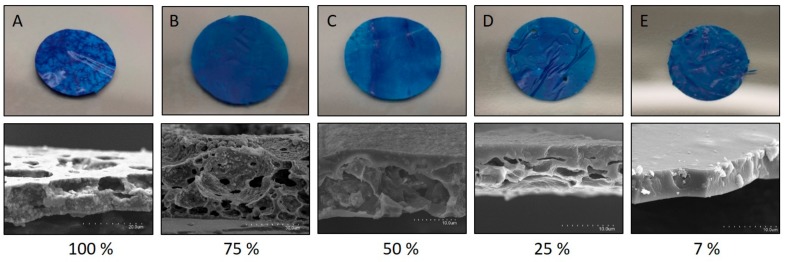
Visual appearance and SEM image of the cryofracture surface of the films obtained from a 7% EVOH solution with the incorporation of nanoparticles of TiO_2_ and glycerol at various concentrations with respect to the mass of polymer. Top images are photos of 1 cm diameter sensors corresponding to the SEM images below.

As described in the experimental section, self-standing films were obtained by casting onto white PE film. The first attempts, developed from 7% EVOH dispersions containing 100% of glycerol, 100% of TiO_2_ and 2.65% of MB, resulted in highly heterogeneous films, with white and blue areas indicating bad integration of the components and fading by contact. Nevertheless, the films were flexible and easy to handle. Apparently the components were too concentrated, so the TiO_2_ and glycerol concentrations were reduced by the same proportion. Figure 1 includes images of the sensors obtained with the following concentrations of TiO_2_ and glycerol: 100%, 75%, 50%, 25% and 7% (with respect to polymer weight).

**Figure 2:**
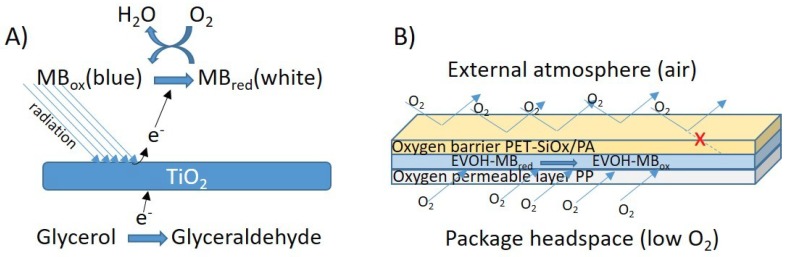

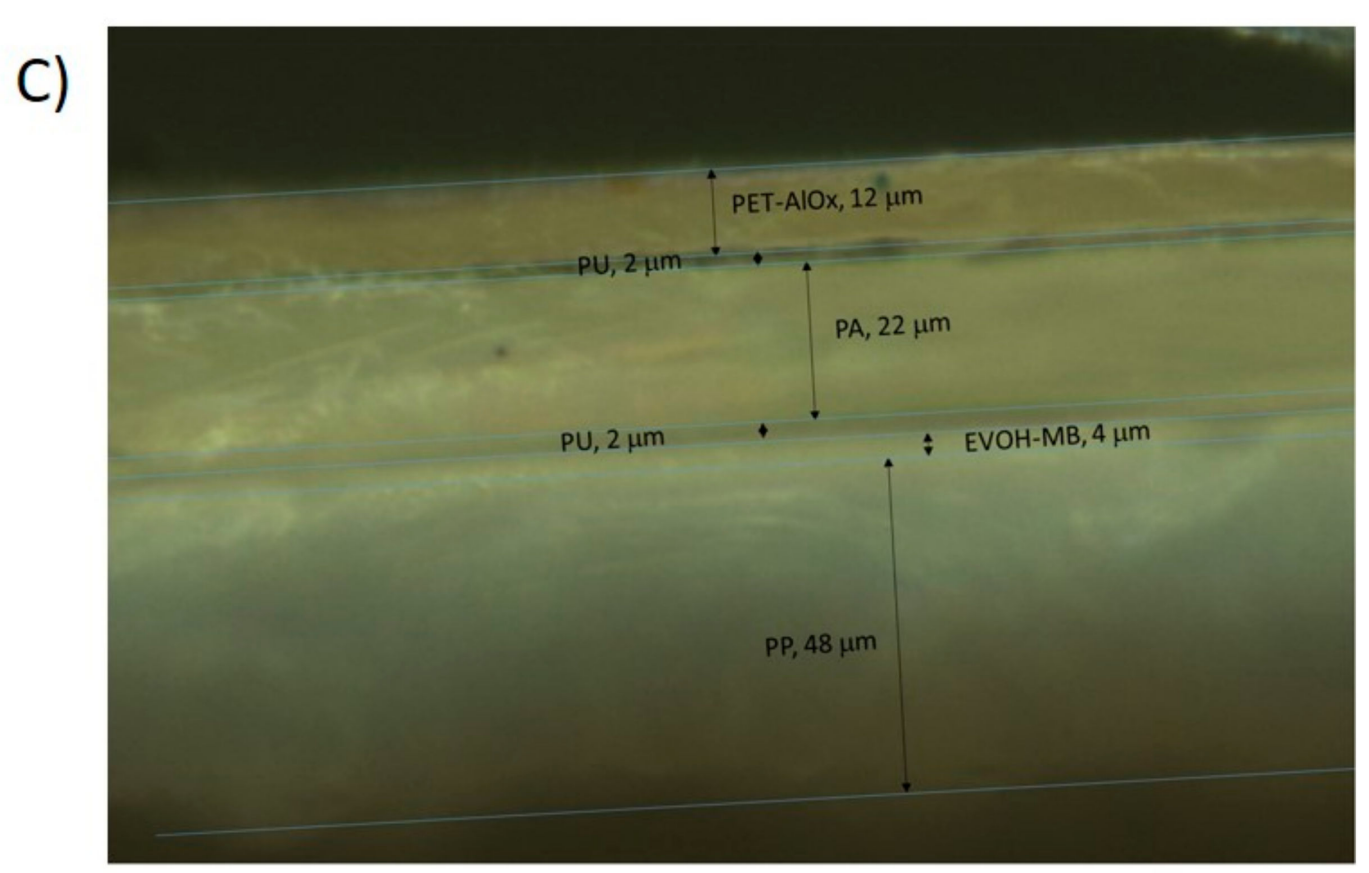
Scheme of the functioning process (**A**) and multilayer assembly (**B**) of the sensor, and Image of the multilayer structure of the oxygen sensor (**C**).

As can be seen in Figure 1, high concentrations of TiO_2_ created discontinuous and heterogeneous materials. In Figure 1A,B, the sensor consisted of TiO_2_ nanoparticles that scarcely adhered to the EVOH polymer, and it contained large voids. However, when the TiO_2_ concentration was reduced to 7% (Figure 1E) the EVOH produced a continuous matrix that contained the nanoparticles uniformly distributed without porosity. This last concentration was selected for validation and further characterization of the sensor. Thus, the final concentrations of the various components of the EVOH films were 7% of glycerol, 7% of TiO_2_, and 0.2% of MB. With this formulation, industrial equipments (an 8-colour flexographic press and an all-in-one laminator) were used to manufacture a multilayer film. A white PP film was coated by flexography with the above varnish formulation on the corona-treated surface, and immediately laminated to a PET-AlOx/PA structure with a polyurethane adhesive. This multilayer has a very high oxygen barrier on the external surface to prevent oxygen from the atmosphere reaching the intelligent coating, and a low-barrier film (PP) to allow oxygen molecules from the food package headspace to reach the sensor [25]. Figure 2 is an overall scheme of the senor functioning description and the multilayer structure assembly. Figure 2C shows a SEM image of a cryofracture surface of the final product with the various multilayer components. The EVOH coating that forms the sensing part of the material, EVOH-MB, is 4 ± 1 µm thick.

### 3.3. Characterization of the Sensor

To test the capacity of the developed material to act as a sensor, the first step was to confirm that in the absence of oxygen the colour changed from blue to white when irradiated. As indicated in the experimental section, circular samples from the various films were cut and packaged under vacuum in high-barrier bags. Then the bags were irradiated with the various devices until a white colour was obtained. With the UV Xe excimer lamp (172 nm), no colour change was detected after 30 min of irradiation. Irradiation with the NIQ 80/36 U Heraeus lamp (253.7 nm) resulted in a slow colour change from blue to white that was complete in 20 min (see Supplementary Materials, Figure S3). Finally, exposure of the sensors to 5 Osram 64,700 halogen lamps resulted in a fast colour change, taking less than 1 min. A video included in the Supplementary Materials shows the colour change in a representative process, in which the sensors are fully white after 50 s of exposure (Figure S4). Therefore this method of sensor activation was preferred and selected. After activation the sensors started to change back towards the initial blue colour, although the evolution was heterogeneous owing to the presence of some residual oxygen in certain parts of the package (despite the vacuum process). In fact, the commercial oxygen analysers inserted in the bags measured oxygen levels ranging from 1 to 3%, remaining almost unchanged during the ten days of testing. Representative images of this process are shown in the Supplementary Materials (Figure S5).

**Figure 3:**
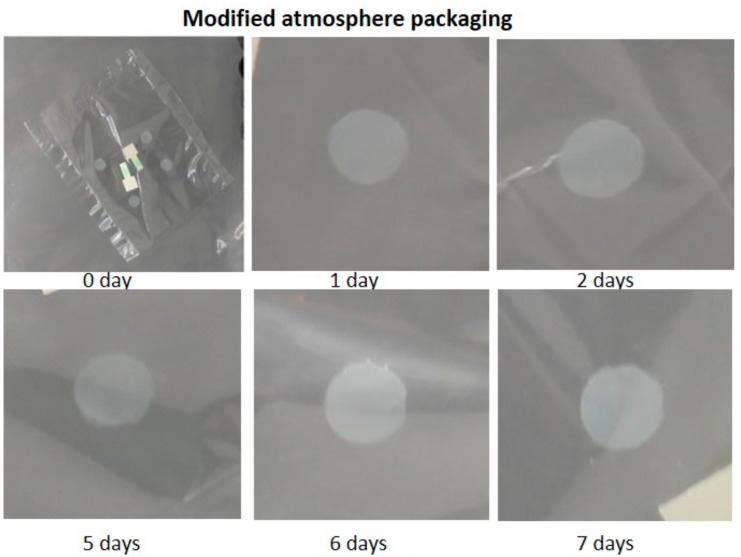
Evolution of activated sensors stored in an inert N_2_ atmosphere.

To reduce this heterogeneity and improve the oxygen evacuation process, sensor samples were inserted in PP bags which were placed in high-barrier bags filled with nitrogen atmosphere. This procedure involves the replacement of atmospheric air by application of a vacuum, injection of nitrogen and sealing of the bag. Following the procedure described in the experimental section, an O_2_ concentration close to 0% was obtained and maintained over a long period of time (>30 days). Figure 3 shows that the colour of the sensors remained white when this type of packaging was used.

Since the objective of the present work was to develop a sensor capable of changing colour in the presence of oxygen, once it had been verified that a colour change occurred when irradiated and maintained in the absence of oxygen, its reversibility in the presence of oxygen was tested. The minimum oxygen concentration and the time required to return to the original blue colour were also analysed.

**Figure 4:**
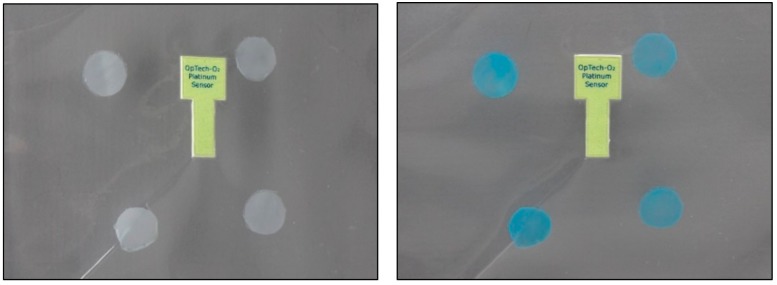
Sensor samples in a PP bag after irradiation (**left**) and 1 h after exposure to air (**right**).

A first simple test consisted in cutting the external bag and exposing the sensors in the PP bag to atmospheric air. All the formulations tested in the optimization of the sensor turned to the original blue colour in less than 1 h, as can be seen in Figure 4, confirming their reversibility. Earlier reports on colour recovery of MB-based sensors showed slower [7,8,11] or similar recovery times [9,14,15], although in all cases the sensing element were directly exposed to air and not as part of a fully developed packaging structure. After verifying that the sensors worked, the minimum amount of oxygen present capable of producing the white to blue change was assessed. For this purpose, sensor samples were packed in MAP using N_2_ as the replacement gas, activated and stored for 48 h in order to verify that the atmosphere was stable and that there was no leakage in the container that could alter the result by increasing the concentration of oxygen. An OpTech oxygen sensor was included as well to record the concentration of oxygen in the bag. Then known quantities of atmospheric air were injected with a syringe, using a self-adhesive septum, and the oxygen concentration and colour measurement were immediately monitored during the first six hours and for the following 15 days, measuring the same parameters daily, since after that time no appreciable changes were observed. The step-change from white to blue (quantitatively determined when the b* values became negative) was established as a reference to mark the development of the colour change.

**Figure 5:**
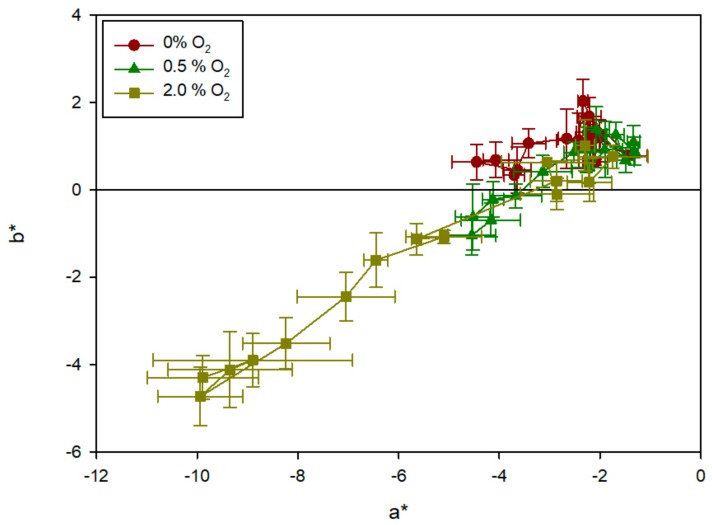
Colour evolution (parameters a* [green-red] and b* [blue-yellow] represented as mean and deviations) of oxygen sensors exposed to various oxygen concentrations after 0, 1, 2, 4, 6 and 24 h, and after 2, 3, 5, 7 and 10 days. The samples evolve from the top right corner to the bottom left corner.

The results obtained showed that the presence of oxygen was responsible for the colour change of the sensors. As can be seen in Figure 5, the sensors in the control bags without oxygen remained white, and thus the b* values were positive throughout the tests. This result is in agreement with the stability showed by previous MB-based sensors in the absence of oxygen [7,8,9,11,14,15]. No significant change was observed and the values remained around a* ≈ –3 and b* ≈ 1, with deviations in the two parameters caused by fluctuations in the measurement itself. At 0.5% of oxygen, the sensors began to change after the first 24 h, with b* becoming negative at day 3. At 2% of oxygen, the colour change was much faster and more intense, with b* reaching negative values after 6 h and full blue development at day 5 (b = –4).

A similar test was carried out with a real food product, specifically with an infant milk powder as a food sensitive to oxidation. For this purpose, 10 milk samples were packed and monitored over 20 days, determining the oxygen content and the colour of the sensors (two in each bag) as indicated in the experimental section. A hexanal concentration analysis was also done at the end of storage.

**Figure 6:**
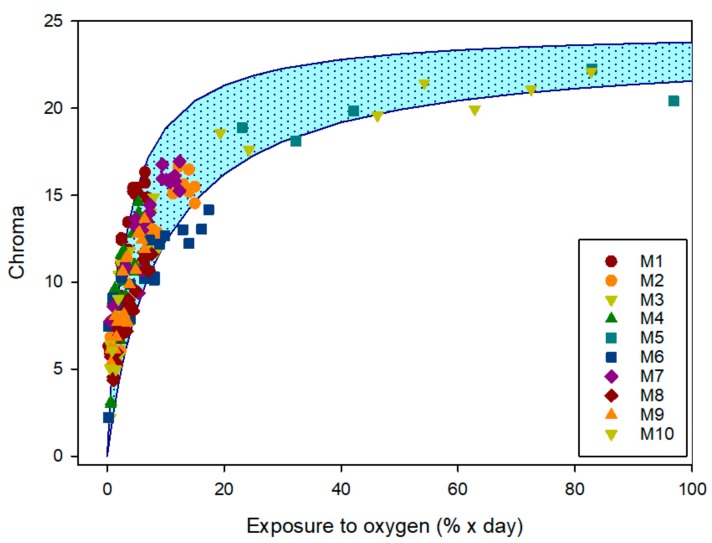
Colour evolution (chroma) of the various sensors as a function of their exposure to oxygen (percentage of oxygen × days, obtained by integration of the oxygen concentration curve vs. time) and theoretical evolution obtained by curve fitting with Langmuir equation (Equation (2)).

In order to easily monitor de evolution of colour, the chroma parameter (or saturation) was determined from the individual (a*,b*) coordinates. The chroma value shows the distance from the coordinate origin in the chromaticity plane, (chroma = (a*^2^ + b*^2^)^1/2^). Since the sensor has a kinetic of change which depends on the percentage of oxygen present and the time of exposure, a parameter called exposure to oxygen was used and it was obtained by integration of the plotted oxygen percentage (%) vs. time (days). Figure 6 shows the evolution of colour (chroma) in the various sensor samples, versus exposure to oxygen. The result presents a clearer evolution of the sensors’ colour, similar to that of an exponential variable that tends to a maximum.

Several expressions were tested to describe the evolution of the sensor colour, and the one that showed the best result was the Langmuir equation:

(2) $$f\;=\frac{\left(B_{max}\;\cdot \int_{0}^{t}\left[O_{2}\right]\cdot dt\right)}{\left(K_{d}\;+\int_{0}^{t}\left[O_{2}\right]\cdot dt\right)}$$

The adjustment gives a value of *B*_max_ = 19.6 ± 1.0 and *K*_d_ = 2.9 ± 1.5. In Figure 6, the experimental data and the description obtained by curve fitting are compared, showing great agreement.

**Figure 7:**
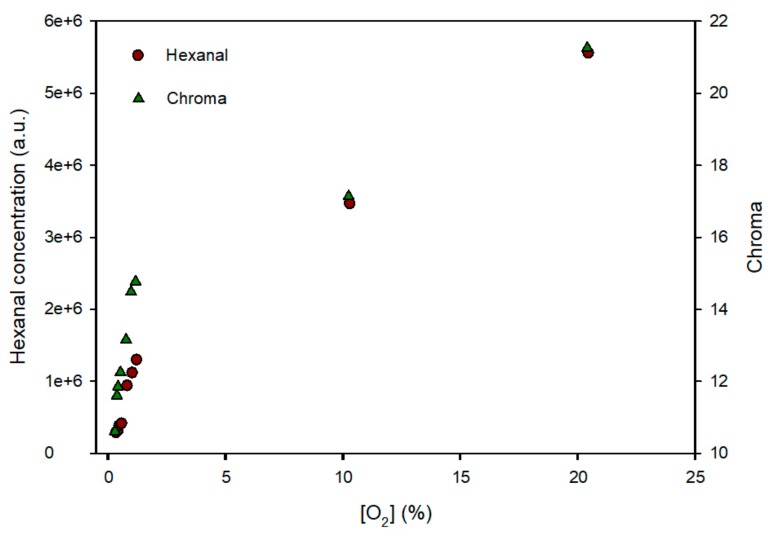
Evolution of the concentration of hexanal (left axis, expressed in chromatographic area units) in dehydrated infant milk bags and colour of the sensor (chroma on the right axis) as a function of oxygen concentration.

To check the evolution of the milk in the MAP bags, an analysis of the hexanal concentration was carried out, as a parameter indicating product oxidation. Figure 7 shows the hexanal concentration (expressed in area units of the chromatographic peak) and the sensors’ chroma at the end of the 20-day storage in the various packages as a function of oxygen concentration. The results reflected the existence of a correlation between the hexanal concentration (a.u.), the oxygen concentration (%) and the chroma of the sensors. As the oxygen concentration increases, the hexanal concentration produced by the oxidation of the milk increases, and also the intensity of the blue colour of the sensors increases as a result of exposure to the oxygen inside the bag. An informal sensory test carried out in the lab consisting in smelling the bag headspace right after bag-opening showed that the samples with 10 and 21% presented a rancid odour confirming that they milk was deteriorated. When these data are observed in detail, it can be deduced that the sensor could indicate the end of the useful life of the food when it reaches a chroma value of 15 since at this value the milk was good and at 17 the milk showed an unpleasant smell. At this intense blue colour, the concentration of oxygen was sufficient to catalyse oxidation reactions in this sensitive food, such as fat peroxidation and hexanal formation. A printed blue band with Chroma = 15 surrounding the sensor will help the consumer to decide whether the product is still edible. For other products, the level of oxygen exposure could be different. Thus, a previous understanding of the oxidation process and a correlation of the product quality with the sensor chroma value would be useful to print a blue band with the colour intensity that indicates end of consumption period. The visibility of the sensor is excellent since the sensor presented a chroma change of 20 units (from the white to the blue colour and it has been reported that a colour change above 3 colour units in the CIE Lab space is readily perceived by human eye [26].

### 3.4. Release of MB from Sensor Structure

Although there is no specific migration limit for MB, an analysis of potential release from the sensor was carried out since this colorant is not included in the list of food colorants or additives. Bags of the film containing 3% acetic acid solution (v/v) as acid food simulant, and 10% ethanol solution (v/v) as aqueous non-acidic food simulant, were prepared and stored for 10 days at 40 °C. Also, bags filled with distilled water were placed in boiling water for 15 min. The simulants were analysed by spectroscopy, following the procedure described in the experimental section. No trace of MB was observed in any sample. According to the calibration prepared, these results indicate that MB migration, if any, is below 10 µg/L. Considering that a real sensor will not cover the whole surface of the bag (200 cm^2^) but only 2 cm^2^, the potential release would be 0.1 µg/L, a quantity without any potential risk. Japan has established a maximum residue limit of 10 µg/kg for methylene blue in aquatic products, because it is used as a replacement for other antifungal dyes in aquaculture [27].

## 4. Conclusions

A sensor based on methylene blue, glycerol, titanium dioxide, and EVOH has been prepared and optimized for the detection of oxygen at concentrations as low as 0.5%. This sensor can be implemented industrially by flexography, and with this technique a flexible structure valid for lids of trays or cups or for bags was manufactured industrially with the following formulation: PET-AlOx/PA/PU/MB-EVOH/white PP (left layers are external, right layers are internal. The external layers provide an oxygen barrier for the packaging system, and white PP allows oxygen from the headspace to reach the sensor and be detected. The cost of the sensor can be optimized by printing the sensor as dots. No migration of MB could be detected, making this intelligent packaging system valid even for products that have to be thermally treated in the package.

## Figures and Tables

**Table 1:** Effect of ethylene-vinyl alcohol copolymer (EVOH) concentration (w/v) on viscosity of polymer solutions, and of TiO_2_ concentration (w/w) on viscosity of dispersions containing 7% EVOH, measured in a no. 4 Ford cup.

EVOH Solutions in PrOH/H_2_O		TiO_2_ Dispersions in 7% EVOH Solutions	
Concentration	Time (s)	Concentration	Time (s)
7%	22.0 ± 0.5^a^	0%	22.0 ± 0.5^a^
9%	37.0 ± 0.5 ^b^	25% TiO_2_	21.8 ± 0.5^a^
11%	55.3 ± 0.6^c^	50% TiO_2_	22.0 ± 0.5^a^
13%	94.7 ± 1.2^d^	100% TiO_2_	21.7 ± 0.5^a^

^a,b^ different letters are indicative of significant differences between average values.

**Table 2:** Values of the Ostwald–de Waele parameters (***K***, consistency, and ***n***, flow behaviour index) for the EVOH solutions and the EVOH/TiO_2_ dispersions at various concentrations.

EVOH Solutions in PrOH/H_2_O			TiO_2_ Dispersions in 7% EVOH Solutions		
Conc.	*K* (Pa·s)	*n*	Conc.	*K* (Pa·s)	*n*
7%	0.057 ± 0.004^a^	0.996 ± 0.006^a^	0% TiO_2_	0.057 ±0.004^a^	0.996 ± 0.006^b^
8%	0.090 ± 0.052^b^	0.992 ± 0.004^a^	25% TiO_2_	0.088 ±0.000^b^	0.967± 0.008 ^a^
9%	0.139 ± 0.013^c^	0.983 ± 0.008^a^	50% TiO_2_	0.090±0.017 ^b^	0.968± 0.023 ^a^
11%	0.237 ± 0.002^d^	0.997 ± 0.001^a^	100% TiO_2_	0.086±0.004 ^b^	0.974± 0.022 ^a^
13%	0.431 ± 0.008^e^	0.991 ± 0.003			

^a,b^ different letters are indicative of significant differences between average values.

## Supplementary Materials

The following are available online at https://www.mdpi.com/1424-8220/19/21/4684/s1, Figure S1: Shear stress vs shear rate values obtained in the rheological test of EVOH hydroalcoholic solutions at polymer concentrations ranging from 7 to 13% (w/v), Figure S2. Shear stress vs shear rate values obtained in the rheological test of 7% (w/v) EVOH hydroalcoholic solutions including various amounts of TiO_2_, from 0 to 100% (w/w of dry polymer), Figure S3. Colour change of the sensor samples after various times of exposure to the irradiation of a NIQ 80/36 U lamp, Figure S4. Video showing the colour turn of the sensors exposed to halogen lamps, Figure S5. Representative colour evolution of activated sensors under vacuum, Video S1: Video showing the colour turn of the sensors exposed to halogen lamps.
